# Monitoring Autophagy in Human Aging: Key Cell Models and Insights

**DOI:** 10.31083/FBL27091

**Published:** 2025-03-20

**Authors:** Tatiana M. Moreno, Jose L. Nieto-Torres, Caroline Kumsta

**Affiliations:** 1 Graduate School of Biomedical Sciences, Sanford Burnham Prebys Medical Discovery Institute, La Jolla, CA 92037, USA; 2 Department of Biomedical Sciences, School of Health Sciences, Universidad Cardenal Herrera-CEU, CEU Universities, 46115 Valencia, Spain; 3 Development, Aging and Regeneration Program, Sanford Burnham Prebys Medical Discovery Institute, La Jolla, CA 92037, USA

**Keywords:** autophagy, aging, human cell models, biomarkers, chronic age-related diseases, peripheral blood mononuclear cells (PBMCs), primary dermal fibroblasts (PDFs), induced neurons (iNs)

## Abstract

Autophagy, a key cellular degradation and recycling pathway, is critical for maintaining cellular homeostasis and responding to metabolic and environmental stress. Evidence for age-related autophagic dysfunction and its implications in chronic age-related diseases including neurodegeneration is accumulating. However, as a complex, multi-step process, autophagy can be challenging to measure, particularly in humans and human aging- and disease-relevant models. This review describes the links between macroautophagy, aging, and chronic age-related diseases. We present three novel human cell models, peripheral blood mononuclear cells (PBMCs), primary dermal fibroblasts (PDFs), and induced neurons (iNs), which serve as essential tools for studying autophagy flux and assessing its potential as a biomarker for aging. Unlike traditional models, these cell models retain age- and disease-associated molecular signatures, enhancing their relevance for human studies. The development of robust tools and methodologies for measuring autophagy flux in human cell models holds promise for advancing our understanding of autophagy’s role in aging and age-related diseases, ultimately facilitating the discovery of therapies to enhance health outcomes.

## 1. Introduction

Aging is a complex and progressive process involving cumulative cellular and molecular changes that lead to a gradual decline in function across various tissues and organs, increasing susceptibility to chronic diseases, including neurodegeneration. Autophagy, a conserved cellular degradation and recycling mechanism plays a critical role in mitigating these age-related changes by maintaining cellular homeostasis and supporting survival. In response to cellular stressors that result in rapid waste accumulation or require cells to obtain nutrients from intracellular recycling pathways, autophagy is upregulated, enabling cells to clear damaged components and recycle resources. However, the efficiency of autophagy declines with age, which contributes to cellular dysfunction and the progression of age-related diseases such as neurodegeneration, highlighting the importance of understanding autophagy in the context of aging.

This review first provides an overview of the autophagy pathway, with a focus on the molecular machinery involved in each autophagic stage. We then discuss evidence of age-related autophagy dysregulation, examining insights from model organisms and existing evidence in humans. Finally, we explore novel human cell models, peripheral blood mononuclear cells (PBMCs), primary dermal fibroblasts (PDFs), and induced neurons (iNs), as promising tools for studying autophagy in the context of aging and chronic diseases. Specifically, we cover autophagy flux in human tissues and cell models, age-related changes in autophagy flux in PBMCs, and the aging signatures retained in PDFs. By highlighting these innovative models and their relevance to human autophagy, this review underscores the importance of robust methodologies for studying autophagy in aging and outlines promising pathways for identifying biomarkers and therapeutic targets.

## 2. Overview of Autophagy

### 2.1 Autophagy Machinery

**Fig. 1.**
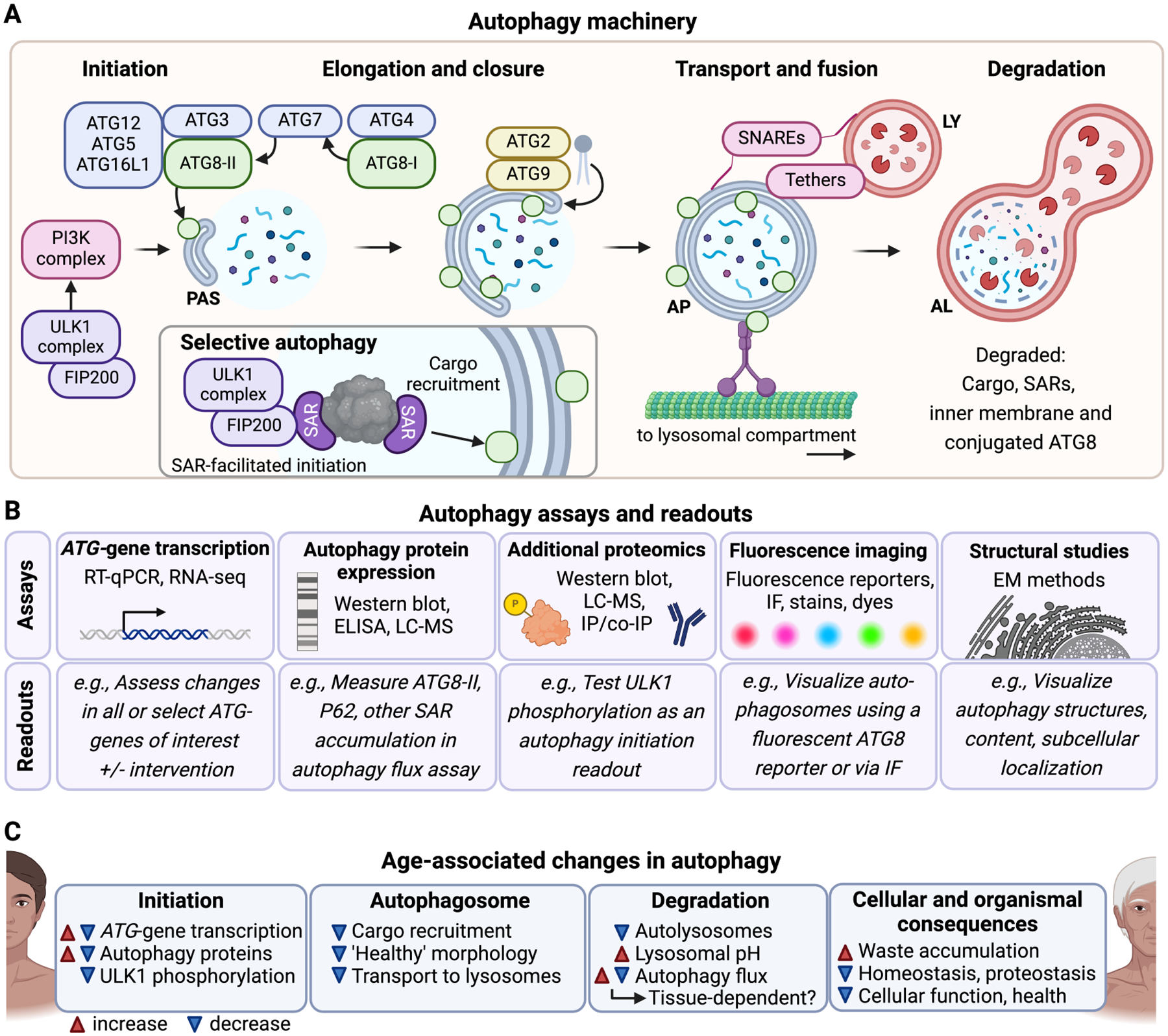
Overview of autophagy: mechanism, assays, and age-related changes in humans. (A) The multi-step autophagy process is depicted in stages: initiation, elongation and closure, transport and fusion, and degradation. In the initiation phase, the Unc-51 Like Autophagy Activating Kinase 1 (ULK1) and Phosphatidylinositol 3-Kinase (PI3K) complexes activate autophagy by recruiting key autophagy-related proteins (ATGs) to the pre-autophagosomal structure (PAS), where phagophore formation begins. During elongation and closure, ATG proteins, such as ATG3, ATG7, and ATG8, facilitate phagophore growth and closure around cargo. ATG8 is represented by green circles attached to both the inner and outer autophagosomal membranes. Selective autophagy involves the association of selective autophagy receptors (SARs) and their cargo with the ULK1 complex. SARs can interact with ATG8 proteins on the inner autophagosomal membrane. The autophagosome (AP) is then transported to the lysosome (LY), where SNAREs and tethers facilitate fusion to form an autolysosome (AL), where cargo is degraded. (B) Various biochemical, imaging, and structural assays are used to investigate different stages of the autophagy process and overall autophagy flux. These include *ATG*-gene transcription assays (RT-qPCR, RNA-seq) to measure changes in gene expression, protein expression assays (Western blot, ELISA, LC-MS) to quantify levels of autophagy proteins like ATG8 or SARs like p62, and additional proteomics methods (e.g., co-immunoprecipitation, IP) for investigating post-translational modifications, such as ULK1 phosphorylation. Imaging techniques, including fluorescent reporters, immunofluorescence (IF), stains, and dyes, allow visualization of autophagosomes, while structural studies (e.g., electron microscopy, EM) provide detailed views of autophagy structures, subcellular localization, and morphology. (C) Experimental evidence indicates that autophagy becomes dysregulated in aging and age-related diseases, with effects seen across different stages of autophagy. Dysregulation may manifest as changes in ATG-gene transcription, autophagosome morphology, lysosomal pH, and overall autophagy flux, contributing to waste accumulation, impaired proteostasis, and cellular dysfunction. Phenotypes that increase with age are indicated with a red arrowhead, and phenotypes that decrease with age are indicated with a blue arrowhead. Some phenotypes can change in either or both directions with age. Figure created in BioRender (https://www.biorender.com/).

Autophagy is the process of delivering cellular cargo to lysosomes for degradation and can be classified into different types, including macroautophagy (non-selective and selective), microautophagy, and chaperone-mediated autophagy (CMA). Macroautophagy involves the sequestration of cargo into double-membrane vesicles called autophagosomes for delivery to lysosomes, whereas in microautophagy and CMA specific cargo are recognized and transported directly from the cytoplasm into lysosomes. Autophagy allows for the degradation of cellular cargo via non-selective and selective mechanisms. Non-selective autophagy, also referred to as bulk autophagy, involves the largely indiscriminate sequestration of cytoplasmic material for degradation [1], whereas selective autophagy mediates degradation of specifically targeted cargo such as protein aggregates and damaged organelles [2]; however, most of the autophagy machinery and process is shared between bulk and selective autophagy. The autophagy process can be divided into several stages: initiation, elongation to closure, transport and fusion, and degradation (Fig. 1A). The initiation phase is regulated by the Unc-51 Like Autophagy Activating Kinase 1 (ULK1) complex. ULK1 phosphorylation, which is inhibited by mechanistic Target of Rapamycin Complex 1 (mTORC1) under nutrientrich conditions and activated by AMP-Activated Protein Kinase (AMPK) during energy stress, leads to ULK1 complex assembly on the cytoplasmic surface of cellular membranes, including the endoplasmic reticulum (ER) [3]. This activates downstream autophagy initiation proteins in the Phosphatidylinositol 3-Kinase (PI3K) complex, stabilizing the initiation structure and promoting the growth of the phagophore, which will mature into a fully formed, double-membraned autophagosome [4]. While the exact mechanisms for lipid acquisition during phagophore growth are incompletely understood, sources may include the ER [5], mitochondria [6], trans-Golgi network [7], and endosomal compartment [8]. Autophagy-related protein (ATG)2 is critical in transferring lipids to the expanding phagophore [9,10], working with ATG9 and ATG18 at the phagophore initiation site to facilitate lipid transfer to the growing phagophore [11]. ATG2 recruits lipids to the cytoplasmic phagophore lipid layer and ATG9 facilitates transport of lipids from the outer to the inner lipid layer [12]. Ankyrin Repeat and FYVE Domain Containing 1 (ANKFY1) may also assist in lipid recruitment from the endosomal compartment [13].

The ATG8 family of proteins plays a crucial role in autophagosome formation and maturation. In humans, there are seven orthologs to yeast ATG8 belonging to two sub-families: Microtubule-Associated Protein 1 Light Chain 3 (LC3A, LC3B, LC3B2, and LC3C) and GABA Type A Receptor-Associated Protein (GABARAP, GABARAPL1, and GABARAPL2) [14]. Throughout phagophore growth, ATG8-family proteins are cleaved by the protease ATG4, which exposes a C-terminal glycine, producing ATG8-I. The cleaved ATG8-I undergoes further modification in a process similar to protein ubiquitination: the E1-like protein ATG7 activates the cleaved ATG8-I and transfers it to the E2-like protein ATG3. Together with the ATG12-ATG5-ATG16L1 complex, ATG8-I is conjugated to phosphatidylethanolamine (PE), forming ATG8-II, and inserted into both the inner and outer membrane of the growing phagophore [15] (Fig. 1A). As the autophagosome matures, ATG8 on the outer membrane helps mediate interaction with dyneins and kinesins, enabling the transport of autophagosomes along the cytoskeleton toward the lysosomal compartment [16]. After the autophagosome fuses with the lysosome, ATG8 on the inner membrane is degraded along with the cargo, while ATG8 on the outer membrane is recycled back into the cytosol [17]. Due to their essential role and because of their incorporation into autophagosomal membranes, ATG8 proteins are commonly used as markers for autophagic activity [18] (Fig. 1B). Immunofluorescence allows the visualization of autophagosomes as ATG8-positive foci using fluorophore-tagged ATG8, providing insights into autophagosome formation and localization. Meanwhile, Western blotting detects the conversion of ATG8-I to ATG8-II by distinguishing their size difference, enabling the quantification of autophagosome-bound ATG8. Both immunofluorescence and Western blot allow the assessment of autophagic flux (the overall rate of autophagy from initiation to degradation) when coupled with autophagy inhibitors [18].

Autophagosome-lysosome fusion, which forms the autolysosome, is mediated by tether and soluble N-ethylmaleimide-sensitive factor activating protein receptor (SNARE) complexes such as the Homotypic Fusion and Protein Sorting (HOPS) complex and STX17-SNAP29 [19] (Fig. 1A). Rab GTPases, including RAB7 and RAB2A in mammals, help guide tether proteins to the surfaces of the autophagosome and lysosome, facilitating fusion [19]. After fusion, the autophagy cargo inside the autolysosome is degraded by lysosomal proteases, hydrolases, and lipases. These enzymes function optimally in the acidic environment of the lysosome, which is maintained by the V-ATPase, a proton pump, which transports protons from the cytoplasm into the lysosomal lumen [20]. This degradative process results in biomolecules such as fatty acids and amino acids, which are then stored in the lysosome or transported to the cytosol, where they can be used for energy production or to synthesize new cellular components [21].

### 2.2 Selective Autophagy

In selective autophagy, autophagy receptors recruit specific cargo, such as ubiquitinated protein aggregates or damaged mitochondria, to the autophagosome [2]. Selective autophagy receptors usually contain an LC3/ATG8-interacting region (LIR) motif and a binding domain for the cargo. They link the recruited cargo to lipidated LC3/ATG8 proteins on the inner autophagosomal membrane, and are eventually degraded in the autolysosome [2] (Fig. 1A). Selective autophagy receptors can also play a critical role in the formation of the autophagosome around target cargo. This is seen in processes like NDP52-mediated xenophagy [22] and P62-mediated aggrephagy [23], where interactions with the ULK1 complex components (including FIP200) assist in forming the autophagosome [24] (Fig. 1A).

Since selective autophagy receptors are recruited to the autophagosome and degraded in the autolysosome, they can serve as autophagy markers (Fig. 1B). Additionally, the accumulation of known autophagy cargos, such as specific proteins or organelles, can also indicate autophagic activity. The most commonly used readout for selective autophagy is currently the accumulation of P62 [18], which indicates turnover of ubiquitin-dependent cargo including aggregates and potentially aberrant mitochondria. The discovery of additional autophagy receptors presents a significant opportunity to develop new assays that could distinguish between different types of selective autophagy and the specific cargo being degraded, providing more comprehensive insights into the types of autophagy occurring in human cells. However, it is important to consider the rate of *de novo* synthesis of the receptors and cargo as well as degradation of cargo via alternative routes such as the protea-some when using these readouts. Since human tissues may have varying needs for the turnover of specific cargo, the development of additional assays for selective autophagy is crucial for accurately assessing autophagic activity in the context of aging, disease, and tissue-specific homeostasis.

### 2.3 Autophagy in Homeostasis and Stress Responses

As a key degradative mechanism, autophagy contributes to intracellular homeostasis under non-stress conditions. The basal rate of autophagy turnover (i.e., autophagy flux) varies across cell type and mitotic stage as observed in primary and immortalized human cell lines and primary rodent cells [25], possibly correlating with metabolic demand. Regardless of basal autophagy flux, non-selective autophagy can be upregulated in response to a wide variety of stressors including starvation, hypoxia, and heat shock to provide essential nutrients and energy and prevent the accumulation of damaged proteins and organelles [1]. Rapid cell proliferation and changes in cell identity associated with organismal development can also lead to upregulation of autophagy [26]. This responsiveness to changing environmental conditions and cellular needs allows cells to ‘clean up’ debris and redirect resources efficiently to promote survival and fitness.

Similarly to bulk autophagy, selective autophagy also occurs under basal conditions in many cell types and can be upregulated in response to cellular stress to combat the accumulation of stress-induced damage [27]. Since selective autophagy relies on autophagy receptors, cells can regulate specific receptors based on their current metabolic demands or the type of stress encountered [27]. Availability and activity of autophagy receptors can be modulated via post-translational modifications and protein-protein interactions including ubiquitination, phosphorylation, acetylation, and dimerization [28]. These functional modifications can be inhibitory or activating. Collectively, these aspects of selective autophagy allow for the tightly regulated activation of selective autophagy subtypes to optimize cellular response to stress conditions.

## 3. Evidence of Age-Related Autophagy Decline

### 3.1 Age-Related Changes in Autophagy: Lessons from Model Organisms

Accumulation of cellular waste, which leads to cellular dysfunction, is a significant contributing factor to aging and chronic age-related diseases such as neurodegeneration, cardiovascular disorders, sarcopenia, and type II diabetes [29]. Autophagy as a key arm of the proteostasis network and an essential degradative pathway for many organelles and other large cytoplasmic structures, has been shown to play a crucial role in mitigating the accumulation of damaged proteins, organelles, and other cellular waste by facilitating their degradation and recycling. Dysfunctional or insufficient autophagy can exacerbate the progression of aging and age-related diseases by allowing toxic aggregates and damaged organelles to accumulate, further contributing to cellular stress, inflammation, and metabolic imbalances [30]. Thus, ‘disabled macroautophagy’ is nowadays considered a primary hallmark of aging, defined as a biological process that not only drives aging but can also be targeted to delay its onset and extend healthspan [31]. Consequently, boosting autophagy or restoring its age-related deficiencies holds a tremendous potential to promote healthspan and longevity [30]. Most of the experimental evidence connecting autophagy and aging comes from diverse model organisms. In *Caenorhabditis elegans*, age-related decline in autophagy-gene transcription and autophagic flux impair the cell’s ability to clear damaged components [32]. Similarly, in *Drosophila*, aging is associated with decreased expression of essential autophagy genes (i.e., ATG2, ATG8a, and ATG18) in neurons, leading to reduced autophagy activity and accumulation of damaged proteins and organelles [33]. Additionally, decreased autophagy contributes to muscle degeneration [34] and impaired intestinal homeostasis in aging flies [35]. In mice, aging is associated with decreased ULK1 phosphorylation (indicative of compromised autophagy initiation) [36], reduced autophagosome formation [37], and in rats, impaired lysosomal activity [38]. LC3/ATG8 and P62 protein expression increases in aged murine motor neurons, but autophagy flux assays suggest that this is due to a block in late-stage autophagy rather than upregulated autophagic activity [39]. Predictably, autophagy targets including cargo known to contribute to age-related disease pathology such as protein aggregates [40] and aberrant mitochondria [41] accumulate in different tissues and organisms with age, and are linked to the onset of chronic age-related diseases. Restoration of autophagy gene/protein expression and activation of autophagy flux through genetic (overexpression of select autophagy genes) [42], transcriptional (overexpression or activation of autophagy-gene transcription factor TFEB) [42], pharmacological (spermidine, metformin, rapamycin supplementation) [43] or behavioral (dietary restriction, exercise) [44] interventions can enhance autophagic function, reduce pathological signatures and improve organismal health outcomes such as cognitive function, motility, stress resistance and lifespan. These consistent findings across species suggest that the age-related decline in autophagy is conserved, and preventing autophagy decline or enhancing autophagy significantly improves cellular health during aging.

However, understanding the age-related decline in autophagy and developing autophagy-enhancing interventions presents several challenges. Autophagy is a multi-step process, and disruptions can occur at any point, including initiation, elongation, or degradation, making it difficult to pinpoint where the process fails during aging and thus, identify a specific therapeutic target. Furthermore, the rates of autophagy decline differ across tissues, adding complexity to analyses. For example, while some tissues may show early defects in autophagosome formation, others may experience issues with lysosomal fusion or degradation [45]. Additionally, blocked autophagic flux can result in an accumulation of autophagosomes, which may be misinterpreted as increased autophagy rather than a failure in the degradation step. This highlights the importance of assessing autophagic flux rather than relying solely on static measurements of autophagosome numbers [18].

These challenges are particularly relevant when translating findings from model organisms to human aging. In humans, autophagy dynamics may be influenced by the complexity of our tissues, the diversity of cell types, and individual genetic and environmental factors. Age-related decline in autophagy may manifest differently across various organs, with certain tissues like the brain or muscles being more susceptible to autophagy dysfunction, contributing to diseases such as neurodegeneration [46] and sarcopenia [47]. Therefore, understanding how specific steps in the autophagy process fail in humans will be crucial for developing therapeutic strategies to restore autophagic function and promote healthy aging.

### 3.2 Age-Related Changes in Autophagy in Humans

Studies investigating age-related changes in the autophagy process in humans are still limited, and the majority rely on measuring expression levels of core autophagy genes and proteins, which do not directly reflect the degradative efficacy and activity of the autophagy pathway. Nevertheless, evidence suggests that age-related alterations in upstream regulators may contribute to autophagy decline. Age-related epigenetic alterations [48] and dysregulation of autophagy-gene transcription factors such as TFEB and FOXOs can lead to decreased expression of autophagy-related genes [42]. For example, *ATG4C* transcripts are reduced in aged human peripheral blood [49], and *BECN1*, *ATG5*, and *ATG7* are reduced in aged human brains [50,51]. Proteomic approaches, including Western blotting and mass spectrometry, also reveal age-related changes in autophagy protein expression, with proteins such as Beclin-1 (BECN1), ULK1, and LC3/ATG8 declining with age in human osteoarthritic chondrocytes [52]. On a structural level, aging and neurodegenerative diseases are associated with an increase in aberrant autophagic structures. Electron microscopy studies have revealed accumulation of stalled immature autophagosomes in aged mouse neurons [37] and rat hepatocytes [53]. Similarly, autophagosome-like structures surrounded by multiple layers of lamina have been observed in induced pluripotent stem cell (iPSC)-derived neurons from Parkinson’s Disease (PD) patients [54]. In these neurons, the contents of autophagosomes are electron-dense (interpreted as un-degraded autophagic cargo) [54], whereas in Huntington’s Disease (HD) patient lymphoblasts and neurons autophagosomes are mostly “empty” (low electron density), possibly due to cargo recognition failure [55]. Lysosomal degradation capacity also declines with age, evidenced by increased luminal pH in PD and Alzheimer’s Disease (AD) post-mortem brain cells, and human PD patient fibroblasts [56]. Together, these data demonstrate how age-related autophagy decline can stem from failures at several stages of the autophagy process [57] (Fig. 1C). Importantly, autophagy flux assays (which measure the overall rate of autophagic activity from initiation to degradation) in PD patient iPSC-derived midbrain neurons reveal a decline in autophagy flux with age [54]. However, rigorous autophagy flux studies in other human tissues are needed to fully characterize autophagic activity during aging.

Despite the compelling data outlined above, evidence indicates that autophagy does not always decline with age and can increase in some contexts. While autophagy is protective against cellular senescence, in already senescent cells, its upregulation can promote the pro-inflammatory senescence-associated secretory phenotype [58]. Cancer cells, especially those in a tumor microenvironment with low nutrient availability, also upregulate autophagy to support their highly active metabolism by recycling cellular components [58]. These findings support a model in which autophagy is dysregulated with age rather than simply inhibited. Further research is needed to disentangle the numerous age-related changes, relevance of cellular states, potential compensatory mechanisms, and additional functions of autophagy-related proteins.

## 4. Autophagy in Human Aging and Chronic Age-Related Disease Models

### 4.1 Autophagy Flux in Human Tissues and Cell Models

**Fig. 2.**
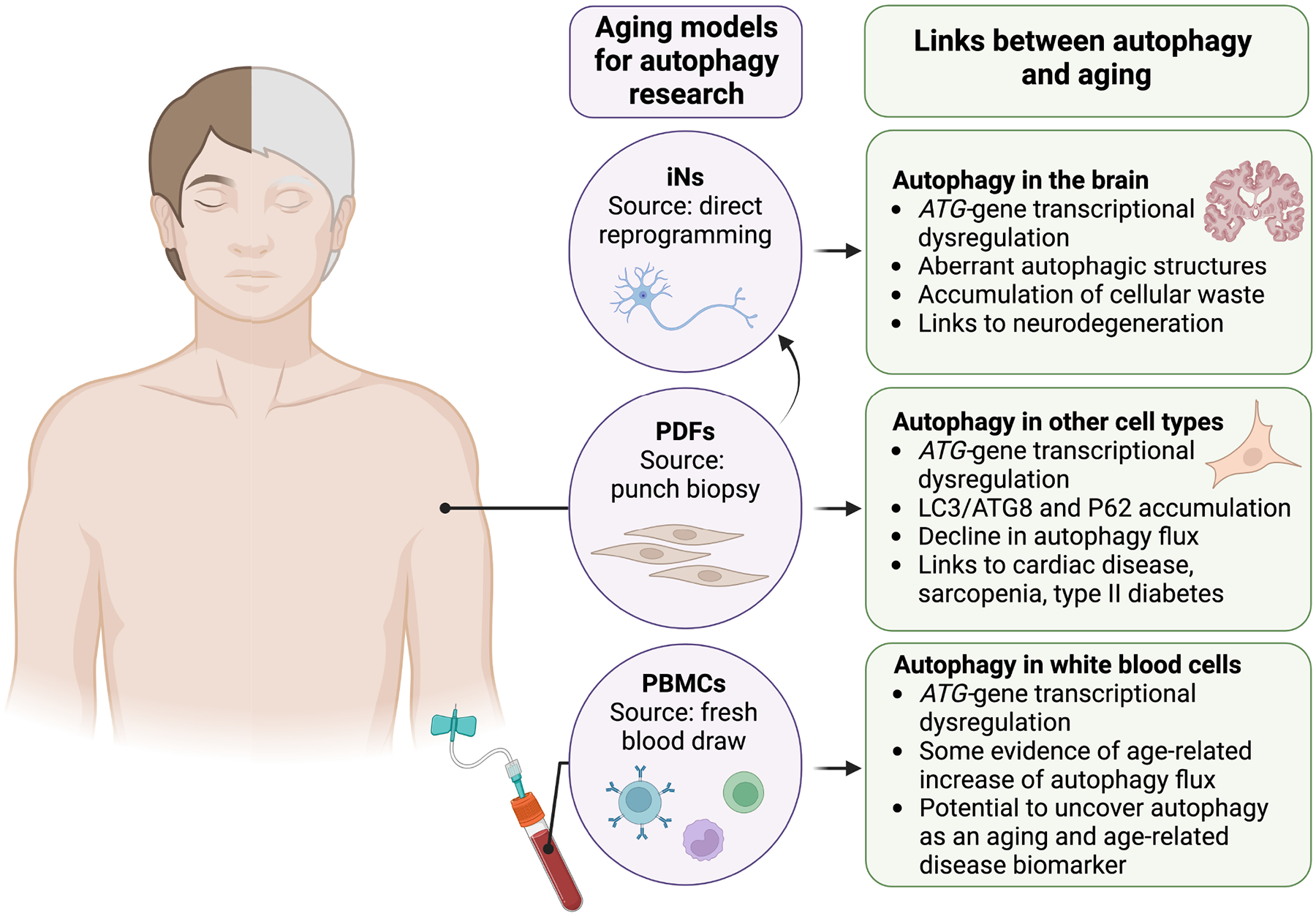
Key human cellular models for aging- and age-related disease-relevant autophagy research. Advances in human cell collection and culture have yielded cell models that largely retain aging- and chronic age-related disease-associated signatures of donors. Three key human cell models are induced neurons (iNs) generated via direct reprogramming, primary dermal fibroblasts (PDFs), and fresh peripheral blood mononuclear cells (PBMCs). Age-related changes in some autophagy readouts have been observed across human tissues (see right panels); the human cell models shown can be used to validate and thoroughly investigate the molecular mechanisms underlying age-related autophagy dysfunction, and test autophagy-enhancing interventions. ATG, autophagy-related. Figure created in BioRender (https://www.biorender.com/).

Until recently, aging-relevant autophagy research in humans and human cell models has been constrained by technical limitations. Accurately assessing overall autophagic activity from initiation to degradation requires autophagy flux assays, as they can distinguish between induced and blocked autophagy. These autophagy flux assays involve treating cells with or without a late-stage autophagy inhibitor, such as a lysosomal V-ATPase inhibitor, for a pre-determined amount of time (usually 1–2 hours), which leads to accumulation of autophagosomes and their cargo. The difference in the number of autophagosomes between treated and untreated cells, assessed by measuring levels of autophagosome-associated proteins such as LC3/ATG8 or counting LC3/ATG8-labeled autophagosomes via immunofluorescent imaging, reveals autophagy flux [18]. Autophagy flux assays cannot be performed in human subjects since late-stage autophagy inhibitors are not safe at the required concentrations. Fortunately, recent technical advances now allow for autophagy flux measurements in fresh human blood samples [59], which more closely resemble *in vivo* conditions, and in cultured human cell models [60] that retain at least some aging [61] and chronic age-related disease-relevant [62] molecular signatures (Fig. 2). These tools may allow for unprecedented advances in understanding the molecular mechanisms regulating autophagy upon aging and in age-related diseases in humans, enabling the testing of autophagy-enhancing interventions to mitigate chronic age-related diseases.

### 4.2 Age-related Changes in Autophagy Flux in Fresh Human Peripheral Blood Mononuclear Cells (PBMCs)

Autophagy-related measures such as autophagy marker expression and autophagosome/lysosome imaging in *ex vivo* human tissues suggest a decline or dysregulation of autophagy associated with aging and chronic age-related diseases [63]. In 2021, a novel autophagy flux assay using fresh human PBMCs was described [59]. This assay involves incubating fresh blood samples under physiological conditions in the presence or absence of a late-stage autophagy inhibitor. PBMCs are then isolated and prepared for protein-based assays (e.g., Western blot or ELISA) or imaging-based assays (e.g., immunofluorescent staining) to assess autophagic activity. While this is not an *in vivo* assay *per se*, maintaining the PBMCs in whole blood in conditions that mimic the human body ostensibly produces data that closely reflects *in vivo* physiology.

This novel, minimally invasive, and relatively simple assay has the potential to facilitate significant advances in our understanding of human autophagy, from basic biology to disease mechanisms and therapeutic interventions. Characterization of autophagy flux in PBMCs from healthy subjects can establish baselines, assess variability, and explore correlations with subject features such as age, sex, and ethnicity. Additionally, studies in patients with diseases may uncover links between autophagy flux and disease phenotypes. Importantly, this assay also allows for intervention studies using behavioral, environmental, or pharmacologic treatments either in human subjects or directly on drawn blood (e.g., *ex vivo* heat exposure), providing a safer alternative for testing treatments not suitable for direct human application. Finally, the accessibility of blood allows for minimal restrictions during study participant recruitment, enabling a broad range of human studies.

Blood is known to carry age-related factors that promote aging in various other tissues. For instance, heterochronic parabiosis experiments in which young and old rodents share a circulatory system have shown that young rodents develop age-related phenotypes and undergo ‘epigenetic and transcriptomic aging’ when exposed to old rodent blood, and conversely, old rodents show physical and molecular signs of rejuvenation [64]. Factors in plasma have been identified as mediators of some of these effects [65], and PBMCs have also been shown to carry age-related molecular signatures [66]. Age-related changes in PBMCs have been of particular interest to the field of immunology, as the immune system is well-known to deteriorate with age and there is evidence for dysfunction associated with aging and chronic age-related diseases in some PBMC subtypes [67]. Intriguingly, autophagy dysregulation has been implicated in some of these immune cell phenotypes [68]. Therefore, it remains to be determined whether autophagy changes with age in PBMCs, how these changes correlate with autophagic activity in other tissues and overall patient health, and whether autophagy flux in PBMCs could serve as a biomarker for related phenotypes [69].

There are some limitations associated with this assay that should be considered during experimental design and data analysis. PBMCs are a heterogeneous population of cells with different functions [70] and could have different baseline and stress-induced autophagy responses. Indeed, autophagy-gene expression varies greatly among PBMCs; *LC3B/ATG8* gene transcription is approximately 3–5 times higher in granulocytes than in monocytes, B-cells, and T-cells [71]. The proportion of each cell type can also vary among individuals and shifts with age [72], which could further complicate assay interpretations. While general markers like LC3B-II/ATG8-II are commonly measured via Western blot or ELISA, these assays do not differentiate between cell types. Further isolating PBMC subpopulations during the PBMC isolation process using magnetic-activated cell sorting (MACS) [73] or after fixing PBMCs using cell-type-specific immunofluorescent staining is possible, albeit at the expense of cell number. Additionally, variability in autophagic activity among subjects could affect statistical power, making it challenging to detect significant differences between groups. Careful consideration of cohort size and participant heterogeneity may be important, especially in studies involving rare diseases or small subpopulations.

Acknowledging both the strengths and shortcomings of this new assay, recent studies have produced intriguing data. In 2023 the Sargeant group measured autophagy flux in PBMCs of 114 study participants aged 35–75 with varying risk levels of developing diabetes, as determined by the Australian Type 2 Diabetes Risk (AUSDRISK) Assessment Tool [74]. Autophagy flux was variable across participants, but the authors found a positive correlation between autophagy flux and age (r = 0.29; *p* = 0.0015 by Spearman’s correlation) [74]. Specifically, autophagy flux increased slightly yet significantly in participants over 65 years old. This result is unexpected given the extensive evidence of age-related autophagy decline in model organisms and post-mortem human tissues. Should these findings be validated by additional studies, it will be important to investigate potential stress responses or compensatory mechanisms that could lead to increased autophagy in these cells. Moreover, determining whether autophagy flux in PBMCs is representative of autophagy in other tissues should also be a priority. Additionally, the Sargeant group reported moderate correlations between autophagy flux and cardiovascular health, supporting the potential of PBMC autophagy flux as a biomarker for cardiovascular health. The Sargeant and Fourrier groups are currently carrying out a study investigating the effects of dietary behaviors on autophagy [75]. It will be interesting to see whether further correlations to physiological, health, and disease readouts will be possible, and to delve into the molecular mechanisms that may link them to autophagy flux in PBMCs.

In addition, Dr. James J. McCormick has led several studies focused on autophagy in human PBMCs. These studies often utilize LC3 expression and LC3-II:LC3-I ratio as a proxy for autophagic activity, rather than autophagy flux assays. Despite this limitation, McCormick and colleagues’ results are consistent with greater autophagy induction upon environmental stimuli such as heat in younger individuals than in older individuals, both *in vivo* [76] and *ex vivo* [77,78]. They also observed better recovery of autophagy to baseline in healthy individuals than in pre-diabetic individuals [79], and a body heat-dependent autophagy increase upon exercise in young individuals [80]. Furthermore, their research indicates that cultured PBMCs isolated from young individuals immediately after exercise show stronger autophagy induction upon rapamycin treatment compared to older individuals [80]. These preliminary findings suggest significant age- and health-related differences in autophagic responses; however, further validation with autophagy flux assays is needed, highlighting the importance of standardizing this assay to ensure findings are reproducible and comparable across laboratories.

### 4.3 Primary Dermal Fibroblasts (PDFs) Retain Aging Signatures

Primary dermal fibroblasts (PDFs) are increasingly used in aging research due to their accessibility via skin punch biopsies and their retention of age- and chronic age-related disease-associated signatures [62,81–83], unlike immortalized cell lines or iPSC-derived cells. This characteristic makes them valuable for aging-relevant mechanistic studies and the development of therapeutics. While other primary cells such as cultured post-mortem [84] or resection-derived [85] brain cells are also used, their availability is often limited to post-mortem donations or surgical biopsies, making PDFs a more practical choice for widespread research applications.

Epigenetic changes are a key molecular feature of aging cells, with specific epigenetic signatures being linked to both chronological and biological age [86], and forming the basis of highly accurate predictive epigenetic clocks [87]. These epigenetic changes significantly modify chromatin accessibility and gene expression, leading to altered cell function and even loss of cell identity [88]. Importantly, in contrast with iPSCs and iPSC-derived cells in which the epigenetic landscape is largely ‘re-set’ to a development stage-like status [89], cultured PDFs from aged donors largely retain age-related epigenetic signatures and epigenetic aging seems to be accelerated in cultured cells [81]. Furthermore, PDFs display altered transcriptomes [90] and proteomes [91] consistent with known age-related trends.

These findings support the idea that the behavior and metabolism of PDFs more accurately parallel *in vivo* cell behavior in humans than non-primary cell types. Moreover, PDFs from older donors also retain phenotypes associated with chronic age-related diseases. For instance, PDFs from HD patients spontaneously produce huntingtin (HTT) aggregates, whereas when the same PDFs are de-differentiated into iPSCs and then re-differentiated back into dermal fibroblasts, the new cells no longer produce HTT aggregates [62]. Thus, PDFs likely more accurately reflect molecular mechanisms relevant to aging and chronic age-related diseases than models that have undergone more extensive manipulation. However, it is important to note that increased passage numbers in culture can diminish the age-related differences between PDFs obtained from young and old individuals [92]. Moreover, prolonged culturing can lead to cellular senescence or selection for subpopulations of cells that proliferate more rapidly, which may not accurately represent the aging phenotype. This limitation underscores the necessity of using low-passage PDFs and carefully monitoring their aging characteristics during experiments.

Some autophagy studies have been carried out in PDFs from young and older donors; however, these have yielded mixed results. While some evidence supports a decline in autophagy flux in PDFs with age [93], other data indicate no significant differences [60]. Both proteomic [91] and LC3/ATG8 immunofluorescence-based [94] studies reveal an increase in select autophagy protein expression, although, without an autophagy flux assay, it is not possible to determine whether autophagy is activated or autophagic degradation is blocked in these cells. Further investigation is needed to determine the source of the conflicting data. Some possibilities include the area from which the skin punch biopsy was taken (e.g., highly sun-exposed skin versus typically shaded skin) [95], donor genetics, cell culture conditions, autophagy flux assay protocols, and number of passages in culture. Standardization of protocols for autophagy flux assays, including consistent methodologies across studies, will be crucial for reducing variability and ensuring more reliable, reproducible results. Despite these early challenges, the overall findings suggest PDFs show promise as a model in which to study the cellular mechanisms involved in aging and chronic age-related diseases including autophagy.

### 4.4 Directly-Reprogrammed Neurons (iNs) for Autophagy and Neurodegeneration Research

Originally, cellular reprogramming required the generation of iPSCs and subsequent differentiation into the desired cell type. While iPSC-derived cells are valuable for many research areas and offer benefits like unlimited replication and diverse differentiation potential, they are usually not appropriate models for aging research. As discussed in the previous section, de-differentiation largely erases age and disease-related molecular signatures, limiting the relevance of data obtained from iPSCs and iPSC-derived differentiated cells [61]. Recent advances now allow for direct reprogramming of cells, bypassing pluripotency. Induced neurons (iNs) can be directly reprogrammed from non-neuronal cells such as PDFs [96] and various blood cells [97]. While induced cell types are still being characterized, PDF-derived iNs appear to largely retain age- and disease-related molecular signatures including epigenetic marks and transcriptomes [61,62]. Age- and chronic age-related disease-associated functional phenotypes are also retained. For example, iNs derived from PDFs of older adults display impaired nucleocytoplasmic compartmentalization [61]. Similarly, iNs derived from PDFs of AD patients show age-related epigenetic marks and appear to shift towards a non-neuronal [98] and pro-inflammatory transcriptome [99], consistent with loss of cell identity and senescence. Additionally, iNs derived from PDFs of HD patients display similar transcriptomic changes associated with loss of neuronal cell identity [100].

To our knowledge, autophagy flux has not been characterized in iNs from healthy young and aged donors; however, some studies have investigated autophagy in iNs from donors with neurodegenerative diseases. iNs from HD patients show impaired autophagy driven by a decrease in autophagosome transport [101], and iNs from PD patients show nearly completely blocked autophagy flux [102]. It will be interesting to test whether autophagy flux is also decreased in iNs of healthy aged donors, and if so, how the extent of decline compares to phenotypes in iNs derived from neurodegeneration patients. Moreover, these cell models may help discern the role of autophagy decline in the onset of neurodegeneration versus the contribution of neurodegenerative pathology to autophagy inhibition, and finally, facilitate testing of potential therapies to mitigate neurodegeneration via autophagy enhancement.

Induced neurons (iNs) as models for autophagy, aging, and neurodegeneration carry some limitations due to their post-mitotic nature, requiring new iNs to be induced for each experiment, which can lead to batch variability. The source cells, such as PDFs, also have limited expansion and replication capacity. Additionally, genetic and epigenetic variability between source cell donors and even related to the specific location from which cells are harvested could impact cellular genetics, epigenetics, and therefore, experimental results. Despite these challenges, iNs are currently among the best cell models for studying the molecular mechanisms of aging and neurodegeneration since they appear to closely mirror *in vivo* neuron behavior and allow for patient-specific research to be performed.

## 5. Conclusions: Current Limitations and Future Directions

Autophagy plays a pivotal role in maintaining cellular homeostasis, and is crucial for understanding the drivers of aging and chronic age-related diseases. Delineating the molecular details of autophagy will be essential for developing strategies to enhance autophagic activity, potentially alleviating various chronic age-related diseases and contributing to improved health outcomes. While studies correlating autophagic activity with longevity and aging have been extensively characterized in animal models, human studies have been constrained by limited experimental techniques. These studies have often relied on partial readouts of the autophagy process, such as transcriptional levels of core autophagy genes and autophagy protein expression, which do not capture the dynamic, multi-step nature of autophagy. Nevertheless, autophagy decline is proposed as a biomarker for aging, and the development of new cellular models is critical for better understanding this relationship.

Autophagy flux assays, which provide a comprehensive assessment of autophagic activity from initiation to degradation, are the gold standard for analyzing autophagy status. However, applying these assays in humans has been challenging due to the need for chemical treatments and processing of live tissue and cellular samples. In this review we present three complementary cellular models to monitor autophagy status in humans: PBMCs, PDFs, and iNs. Patient-derived PBMCs and PDFs can be obtained through routine, minimally invasive procedures, allowing autophagic activity within these cells to serve as potential biomarkers in humans, with direct application in aging and age-related disease studies. Unlike autophagy flux studies in mammalian model organisms, initial studies in human PBMCs suggest that autophagy flux may increase with age. These findings underscore the importance of obtaining human-specific autophagy flux data instead of relying on transcriptomic or proteomic data. To validate these potential biomarkers, systematic analyses should be conducted where autophagy gene expression and autophagy flux assays are simultaneously performed in the three cell types discussed. This approach will help identify potential cell type correlations and differences in autophagy readouts across cell types. Additionally, the sensitivity of these cell models to interventions with known effects on autophagy, such as exercise and diet, should be explored. Importantly, since autophagy status is highly responsive to cellular stress and environmental changes, the methodology used to assess autophagy flux, including inhibitor selection, concentration, assay timing, and cell culture/maintenance conditions, should be standardized across cell models to allow for consistent and comparable results across laboratories.

It is also crucial to test whether autophagy status in these human cell models correlates with longevity and healthspan of individuals, as well as with current leading predictors of biological age, such as epigenetic clocks. Moreover, factors like sex, ethnicity, and other genetic factors known to impact longevity may also influence autophagy status. A significant potential limitation of using autophagy readouts in these cell lines as biomarkers is their ability to predict autophagy status in other tissues across the individual. Since autophagy status likely varies between tissues, autophagy readouts from PBMCs, PDFs, and iNs may represent only a limited view. Therefore, correlative studies of autophagy status in these cell types and samples from other tissues from the same individual would be ideal and could be achieved through collaborations with hospitals and tissue biobanks.

Furthermore, indirect measurements of autophagic activity, such as the extracellular secretion of cargo resulting from autophagy malfunction [103], could be combined and correlated with the proposed autophagy flux measurements. Although these analyses are still theoretical, they could in principle, have practical applications in biological fluids such as plasma or saliva, thereby concentrating molecular information from various tissues. Overall, while autophagy has beneficial effects on health and longevity in model organisms, clear molecular evidence in humans is still lacking. The development of new tools and methods for measuring autophagy flux, such as those presented in this review, will pave the way to study the role of autophagy in aging and age-related diseases and facilitate the discovery of interventions to promote healthy aging.

## Abbreviations

PBMCs: peripheral blood mononuclear cells
PDFs: primary dermal fibroblasts
iNs: induced neurons
CMA: chaperone-mediated autophagy
ULK1: Unc-51 Like Autophagy Activating Kinase 1
mTORC1: mechanistic Target of Rapamycin Complex 1
AMPK: AMP-Activated Protein Kinase
ER: endoplasmic reticulum
PI3K: Phosphatidylinositol 3-Kinase
Atg/ATG: autophagy-related gene/protein
ANKFY1: Ankyrin Repeat and FYVE Domain Containing 1
LC3: Microtubule-Associated Protein 1 Light Chain 3
GABARAP: GABA Type A Receptor-Associated Protein
PE: phosphatidylethanolamine
SNARE: Soluble NSF Attachment Protein Receptor
HOPS: Homotypic Fusion and Protein Sorting
STX17: Syntaxin-17
SNAP29: Synaptosomal-Associated Protein-29
RABs: Ras-Related Proteins
V-ATPase: Vacuolar-Type ATPase
LIR: LC3/ATG8-Interacting Region
P62: Sequestrome-1
CCT2: Chaperonin Containing TCP1 Subunit 2
FAM134B/RETREG1: Reticulophagy Regulator 1
NCOA4: Nuclear Receptor Coactivator-4
NDP52: Nuclear Dot Protein-52
FIP200: FAK Family-Interacting Protein of 200 kDa
TFEB: Transcription Factor EB
FOXO: Forkhead Box O
BECN1: Beclin-1
iPSCs: induced pluripotent stem cells
PD: Parkinson’s Disease
HD: Huntington’s Disease
AD: Alzheimer’s Disease
ELISA: enzyme-linked immunosorbent assay
MACS: magnetic-activated cell sorting
AUDRISK: Australian Type II Diabetes Risk
HTT: Huntingtin
PAS: pre-autophagosomal structure
SARs: selective autophagy receptors
AP: autophagosome
LY: lysosome
AL: autolysosome
(co)-IP: (co)-immunoprecipitation
IF: immunofluorescence
EM: electron microscopy
